# 800 Immediate/Ultra-Early v. Early Burn Excision: A Systematic Review of Surgical Outcomes

**DOI:** 10.1093/jbcr/irac012.349

**Published:** 2022-03-23

**Authors:** Tomer Lagziel, Sophie L Cemaj, Laura M Mafla, Alexander K Karius, Charles S Hultman

**Affiliations:** Johns Hopkins University School of Medicine / Sackler School of Medicine, New-York Program, Tel-Aviv University, Rockville, Maryland; University of Nebraska Medical Center, Omaha, Nebraska; The Johns Hopkins University School of Medicine, Baltimore, Maryland; The Johns Hopkins University School of Medicine, Baltimore, Maryland; Johns Hopkins University School of Medicine, Baltimore, Maryland

## Abstract

**Introduction:**

This is a systematic review which seeks to establish if immediate/ultra-early excision (immediate: < 24 hours, ultra-early: 24 - 72 hours) and grafting is better or equivalent to early excision and grafting (early: 72 hours - 6 days) in adults with major burns. The concept of early excision and grafting, as opposed to late excision (late: >7 days), was introduced by Cope et al. and later popularized by Janzekovic in the 1970s when she introduced the concept of tangential excision. Delaying excision 24 to 48 hours has previously been thought to allow resuscitation and correction of physiologic derangements to optimize outcomes. However, timing for excision and grafting is subject to debate. The outcomes of interest include mortality, length of stay, complication rates, wound healing time, infection rates, physiologic demand, blood loss, and resting energy expenditure.

**Methods:**

In this systematic review, we searched PubMed, Embase, CINAHL, Cochrane, Web of Science, and Scopus for studies that compared outcomes and complications between burn patients with ultra-early and early excisions. From this search, we screened 4235 articles. Through our selection criteria, five articles focusing on timing of burn excision were selected for systematic review.

**Results:**

Five studies observing a total of 382 burn patients, published between 1995 and 2016, were included. All five studies are cohort studies, three were prospective studies while two were retrospective chart reviews. Two studies showed decreased length of stay with immediate/ultra-early excision (Still, Keshavarzi) and decreased time to healing with immediate/ultra-early excision (Guo, Lu). One study demonstrated decreased infection and mortality in ultra-early excision (Keshavarzi). One study demonstrated decreased resting energy expenditure in the ultra-early excision group (Gao). One study showed a decrease in blood transfusion in the immediate/ultra-early excision group (Guo). Both the Guo and Gao studies suggest that concerns over excision during the burn shock period may be unfounded provided that the patient is adequately resuscitated.

**Conclusions:**

Studies investigating the immediate/ultra-early excision of burns tend to show improved outcomes for adults with major burns. It is difficult to attain conclusive data due to the lack in overlap of reported outcomes in modern studies. More studies are needed which compare outcomes in adults with major burns between immediate/ultra-early excision and early excision.

